# Loneliness and biological responses to acute stress in people with Type 2 diabetes

**DOI:** 10.1111/psyp.13341

**Published:** 2019-01-28

**Authors:** Ruth A. Hackett, Lydia Poole, Elizabeth Hunt, Laura Panagi, Andrew Steptoe

**Affiliations:** ^1^ Department of Behavioural Science and Health University College London London UK

**Keywords:** cortisol, inflammation, loneliness, stress responses, Type 2 diabetes

## Abstract

Loneliness is linked with all‐cause mortality and coronary heart disease. Altered neuroendocrine and inflammatory responses to stress constitute potential pathways linking loneliness and ill‐health. Stress responsivity is modified in people with Type 2 diabetes, but it is unclear whether loneliness influences biological stress responses in this population. We assessed interleukin‐6 (IL‐6), interleukin‐1 receptor antagonist (IL‐1RA), monocyte chemoattractant protein‐1 (MCP‐1), and cortisol responses to acute stress in 135 people with Type 2 diabetes. Loneliness was measured used the Revised UCLA Loneliness Scale. Loneliness was inversely associated with cortisol output poststress (*B* = −4.429, *p* = 0.019) independent of age, sex, education, marital status, body mass index, and smoking. Lonelier individuals had raised MCP‐1 concentrations 75 min poststress independent of covariates (*B* = 0.713, *p* = 0.022). No associations between loneliness and IL‐6 or IL‐1RA concentrations were detected. These results suggest that loneliness is associated with disturbances in stress responsivity in people with diabetes, and the impact of loneliness on health in people with diabetes may be mediated in part through dysregulation of inflammatory and neuroendocrine systems. Future research is required to understand if such changes increase the risk of poorer outcomes in this population.

## Abstract 2

Loneliness is a predictor of poorer physical health outcomes. In healthy samples, loneliness is associated with altered biological responses to laboratory stress. Here, we demonstrate for the first time the link between loneliness and stress responses in people with diabetes. Our findings suggest that loneliness is linked with stress‐related biological disturbance in this population. Specifically, we detected blunted cortisol stress responses and raised basal monocyte chemoattractant protein‐1 levels poststress in lonelier individuals with diabetes. These results offer the possibility that the influence of loneliness on health in people with diabetes may be partially mediated through neuroendocrine and inflammatory dysregulation.

## 1 INTRODUCTION

Loneliness is a distressing emotion that occurs when there is a perceived imbalance between one’s social needs and the quality of one’s social relationships (Hawkley & Cacioppo, 2010). In the United Kingdom, loneliness is a relatively common experience, with 21% of adults reporting feeling lonely some of the time and an additional 6% reporting ongoing chronic loneliness (Victor & Yang, 2012).

There is increasing recognition of the link between loneliness and negative health outcomes. Results from two meta‐analyses suggest that loneliness is a predictor of all‐cause mortality, with a 22%–26% increased risk of death found in those reporting high levels of loneliness (Holt‐Lunstad, Smith, Baker, Harris, & Stephenson, 2015; Rico‐Uribe et al., 2018). Loneliness has also been linked with the onset of coronary heart disease (CHD) (Valtorta, Kanaan, Gilbody, Ronzi, & Hanratty, 2016).

In light of these findings there has been interest in identifying biological mechanisms linking loneliness with ill‐health. The neuroendocrine system, encompassing the hypothalamic‐pituitary‐adrenocortical (HPA) axis, is hypothesized to be one pathway through which loneliness operates to impact health (Cacioppo, Cacioppo, Capitanio, & Cole, 2015). Cortisol, the end product of the HPA axis, is characterized by a distinctive diurnal patterning with concentrations highest in the mornings, progressively declining over the course of the day (Adam & Kumari, 2009). Loneliness has been linked with an elevated cortisol awakening response (Adam, Hawkley, Kudielka, & Cacioppo, 2006; Doane & Adam, 2010; Steptoe, Owen, Kunz‐Ebrecht, & Brydon, 2004) and a flattening of the diurnal cortisol slope (Doane & Adam, 2010).

Cortisol is involved in regulating inflammation through glucocorticoid receptor (GR) functions, resulting in the suppression of proinflammatory signaling pathways (Rhen & Cidlowski, 2005). However, there is evidence that GR functioning may be compromised in lonelier individuals, which may have a permissive effect on inflammation (Cole, 2008; Cole et al., 2007). Loneliness has been linked with upregulation of proinflammatory gene expression (Cole, Hawkley, Arevalo, & Cacioppo, 2011), but associations with circulating inflammatory markers have been mixed. Some studies have found increased circulating C‐reactive protein (CRP) levels (Cole et al., 2007; Nersesian et al., 2018), whereas others have found no association (McDade, Hawkley, & Cacioppo, 2006; Mezuk et al., 2016; Shankar, McMunn, Banks, & Steptoe, 2011). Similarly, for fibrinogen, some (Mezuk et al., 2016; Nersesian et al., 2018) but not all studies (Shankar et al., 2011) have detected an association between loneliness and raised fibrinogen concentrations. One recent study of 927 adults detected an association between loneliness and raised circulating interleukin‐6 (IL‐6) concentrations (Nersesian et al., 2018).

Psychophysiological stress testing is another research strategy that has been used to investigate the biological correlates of loneliness (Brown, Creaven, & Gallagher, 2018). The advantage of this method is that it allows dynamic biological responses to be studied under controlled conditions (Steptoe & Poole, 2010). To date, three studies have investigated loneliness and cortisol responses to laboratory stress (Edwards, Bosch, Engeland, Cacioppo, & Marucha, 2010; Hackett, Hamer, Endrighi, Brydon, & Steptoe, 2012; Steptoe et al., 2004). The largest of these studies investigated cortisol responses in a sample of 524 healthy individuals and found that loneliness was associated with reduced cortisol stress responsivity in female participants only (Hackett et al., 2012). The other two studies failed to detect an association between loneliness and cortisol stress responsivity (Edwards et al., 2010; Steptoe et al., 2004). Differences in the age of participants included in these studies, as well as differences in other participant characteristics and sample size, may account for these inconsistent results.

The relationship between loneliness and inflammatory responses to stress in healthy individuals has been assessed in three studies (Hackett et al., 2012; Jaremka et al., 2013; Steptoe et al., 2004). In a sample of 240 men and women, loneliness was associated with heightened fibrinogen responses to stress (Steptoe et al., 2004). Heighted inflammatory responses in IL‐6 and interleukin‐1 receptor antagonist (IL‐1RA) were detected in the Hackett et al. (2012) analysis, along with raised monocyte chemoattractant protein‐1 (MCP‐1) values, but only in women. The final study of loneliness and inflammation found that lonelier individuals had higher IL‐6 and tumor necrosis factor‐α (TNF‐α) production by lipopolysaccharide (LPS) stimulated peripheral blood mononuclear cells following acute stress (Jaremka et al., 2013).

These studies have been conducted in general population samples, but loneliness may be a particular issue in Type 2 diabetes (T2D). Cardiovascular disease (CVD) is the leading cause of death in people with T2D (Emerging Risk Factors Collaboration, 2011), and loneliness has a deleterious effect on cardiovascular health (Valtorta et al., 2016). Diabetes can lead to impaired mobility that may reduce opportunities for social contact, and higher levels of depression and reduced social cohesion have been reported among older people with T2D compared with matched individuals without diabetes (Steptoe et al., 2014). A two‐way process may operate, with cardiometabolic factors associated with loneliness potentially contributing to T2D (Hackett & Steptoe, 2017). Ageing and central obesity, which are leading causes of T2D (Danaei et al., 2011), have been associated with increasing loneliness (Whisman, 2010; Yang & Victor, 2011). Cross‐sectional evidence has linked loneliness with raised glycated hemoglobin (HbA1c; O’Luanaigh et al., 2012) and the metabolic syndrome (Whisman, 2010), which are risk factors for T2D. One cross‐sectional analysis of 8,593 Danish participants found an association between loneliness and self‐reported T2D (Christiansen, Larsen, & Lasgaard, 2016), and this has been confirmed in a large Swiss cohort (Richard et al., 2017). To our knowledge, no study has prospectively linked loneliness with later T2D. However, one analysis of 6,839 individuals found that low social network satisfaction was linked with an increased risk of diabetes in male participants only (Lukaschek, Baumert, Kruse, Meisinger, & Ladwig, 2017), while an analysis of a Dutch population cohort reported that socially isolated individuals were more likely to have newly diagnosed and prevalent T2D (Brinkhues et al., 2017).

Psychosocial factors including loneliness have been shown to impact biological responses in the laboratory (Chida & Hamer, 2008). Previous research has shown that hostility, which is characterized as a cynical, angry temperament (Cook & Medley, 1954), exaggerates disturbances in stress responsivity in people with T2D (Hackett, Lazzarino, Carvalho, Hamer, & Steptoe, 2015) and is associated with CVD risk (Chida & Steptoe, 2009). Considering earlier work linking loneliness with cardiovascular outcomes (Valtorta et al., 2016) and disturbances in stress responsivity (Brown et al., 2018), it is plausible that loneliness would also negatively influence stress responses in this population at high risk of CVD in a similar way to hostility (Emerging Risk Factors Collaboration et al., 2010).

To our knowledge, no study has investigated how loneliness impacts stress responsivity in a sample of people with T2D. Epidemiological evidence has linked T2D with altered neuroendocrine and inflammatory biology. The diurnal cortisol rhythm differs in people with T2D compared to controls without T2D (Hackett, Steptoe, & Kumari, 2014), and flattening of the diurnal cortisol slope and raised evening cortisol concentrations are predictive of new onset prediabetes and T2D in initially healthy individuals (Hackett, Kivimäki, Kumari, & Steptoe, 2016). Meta‐analytic reviews have concluded that heightened inflammation is a risk factor for T2D (Wang et al., 2013), and raised inflammation has been noted in people with overt T2D (Pickup, 2004). In the stress laboratory, participants with T2D have been shown to have blunted cortisol responses to stress along with elevated IL‐6 concentrations (Steptoe et al., 2014). We hypothesized that loneliness as a negative psychosocial stressor would cause a further exaggeration of already disturbed stress responses in people with T2D. To test this hypothesis, we investigated the relationship between loneliness and cortisol, IL‐6, IL‐1RA, and MCP‐1 responses to laboratory stress in a sample of individuals with T2D.

## 2 METHOD

### 2.1 Participants

We recruited 135 individuals with T2D (83 men, 52 women) as part of a large trial investigating biological responses to stress and CVD risk (Steptoe et al., 2014). The participants were recruited from diabetic outpatient and primary care clinics in London. Individuals with any history or previous diagnosis of CHD, inflammatory diseases, or allergies were not eligible to enroll. Those with a doctor‐diagnosed or self‐reported mood disorder were excluded due to the links between mood disorders, inflammation, and CVD risk. Subclinical depressive symptoms are considered in our analyses to account for these links. Participants were instructed to avoid taking anti‐inflammatory and antihistamine medication for 7 days prior to testing. Additionally, participants were asked to avoid alcohol and vigorous exercise in the previous evening, and caffeinated beverages and smoking for at least 2 hr before the laboratory session. If participants reported any symptoms of a cold or other infection on the day of testing, they were rescheduled to an alternate time. Full written informed consent was given by all participants, and ethical approval was granted by National Research Ethics Service.

### 2.2 Psychological measures

The revised UCLA Loneliness Scale (Russell, Peplau, & Cutrona, 1980) was used to assess loneliness in the study. This scale was completed as part of a questionnaire battery before the laboratory session. The questionnaire has 20 items, which were rated on a four‐point self‐report Likert scale from 1 = *never* to 4 = *often*. Total scores ranged from 20 to 80 and were calculated by summing all responses, with higher scores indicating greater loneliness. The internal consistency (Cronbach’s alpha) of the scale was 0.94 in this sample. Single‐item subjective stress ratings were given by participants before and after the stress tasks (immediately following the tasks, then 20, 45, 75 min later). The rating was on a 7‐point scale with higher values indicating greater stress. Depression was measured using the 20‐item Center for Epidemiologic Studies Depression Scale (CES‐D), with higher values indicating greater depressive symptomology (Radloff, 1977). For the purposes of this analysis, we removed the CES‐D item on loneliness leaving 19 items. The Cronbach’s α of the scale was 0.869.

### 2.3 Other measures

Participants provided information on age, sex, smoking status (yes/no), marital status, and level of education. Marital status was coded into three categories, where 1 = *single*, 2 = *married*, and 3 = *divorced or separated or widowed*. Education was coded as no qualifications, up to GSCE level (junior high school certificate), A levels (high school certificate), or university degree and above. Anthropometric measurements were taken during the study using standardized techniques, and body mass index (BMI) was calculated from the participants’ height and weight data (kg/m^2^).

### 2.4 Mental stress tasks

Participants completed two 5‐min behavioral tasks designed to induce mental stress, in a randomly allocated order. The mirror tracing task involved tracing a star that could be seen only in mirror image using a metal stylus. When the stylus came off the star, a loud beep was emitted by the device and a mistake was registered (Lafayette Instruments Corp, Lafayette, IN). The participants were told that the average person could trace the star five times in 5 min with a minimum of mistakes. The second task was a computerized version of the Stroop color‐word interference task, where target color words (e.g., yellow, green) were successively presented in a different color ink. At the bottom of the screen during the task, there were four names of colors printed in incongruous ink. Participants were asked to press the computer key corresponding to the position at the bottom of the screen of the name of the color in which the target word was presented. These tasks have previously been used in many studies within this laboratory (Hackett et al., 2012) and have shown similar appraisals of involvement and engagement from participants of varied socioeconomic status (Steptoe et al., 2002).

### 2.5 Procedure

Participants were individually tested in the morning or afternoon in a light‐ and temperature‐controlled laboratory. Measurement devices for the assessment of cardiovascular activity (not described here) were attached, and a venous cannula was inserted for blood sample collection. After this, the participant rested for 30 min, then saliva was collected for cortisol analysis, a baseline blood sample was drawn, and a subjective stress rating was taken. The two 5‐min behavioral tasks followed. Blood and saliva samples and subjective stress ratings were taken immediately following these tasks. Post‐task recovery monitoring continued for 75 min, with further saliva samples and subjective stress ratings obtained at 20, 45, and 75 min post‐task. Blood samples were obtained at 45 and 75 min after tasks. Blood samples were not drawn at 20 min post‐task, as changes in inflammatory markers have generally been reported 40 min and later following acute stress (Marsland, Walsh, Lockwood, & John‐Henderson, 2017).

### 2.6 Biological measures

Blood samples were collected in EDTA tubes and immediately centrifuged for 10 min at 2,500 rpm at room temperature. Plasma was removed from the tube, aliquoted into 0.5‐ml portions, and stored at −80°C until analysis. MCP‐1 and IL‐1RA were assayed in duplicate using fluorescent‐labeled capture antibody beads from Millipore (Milliplex Human Cytokine/Chemokine kit, Millipore Corporation, US), while concentrations were determined with Luminex flow cytometer technology from Bio Rad (Bio‐Plex, Hercules, CA). The limit of detection for MCP‐1 was 1.2 pg/ml, and the mean inter‐ and intra‐assay coefficient of variations (CVs) were 12% and 6.1%, respectively. For IL‐1Ra, the limit of detection was 2.3 pg/ml and the mean inter‐ and intra‐assay CVs 6% and 4.6%, respectively. Plasma IL‐6 was assayed using Quantikine high sensitivity, two‐site enzyme‐linked immunosorbent assay (ELISA) from R&D Systems (Oxford, UK). The intra‐ and interassay CVs were 7.3% and 7.7%, respectively, and the sensitivity of the assay ranged from 0.016 to 0.110 pg/ml. Saliva samples were collected using Salivettes (Sarstedt, Leicester, UK) and were stored at −20°C until analysis. Cortisol levels were assessed from these samples using a time‐resolved immunoassay with fluorescence detection, at the University of Dresden. The intra‐ and interassay CVs were less than 8%.

### 2.7 Statistical analysis

Plasma MCP‐1 values were normally distributed, but IL‐6, IL‐1RA, and cortisol were skewed and were log‐*n* transformed for all analyses. The pattern of cortisol output during testing was analyzed using individual values and also by computing cortisol area under the curve (AUC) with respect to ground using procedures described by Pruessner, Kirschbaum, Meinlschmid, and Hellhammer (2003).

Associations between loneliness and participant characteristics were assessed using univariate analysis of variance (ANOVA) for categorical variables and Pearson’s correlations for continuous variables. Responses to mental stress testing were analyzed using repeated measures ANOVA. IL‐6, IL‐1RA, and MCP‐1 were analyzed across four trials (baseline, task, 45 min, and 75 min post‐task). Subjective stress and cortisol were analyzed across five trials (baseline, task, 20 min, 45 min, and 75 min post‐task). Associations with loneliness were analyzed using multiple regression. Multivariable linear regressions on baseline values IL‐6, IL‐1RA, and MCP‐1 and regressions on responses after stress (immediately post‐task, 45 min post‐task, 75 min post‐task) were carried out using raw values. We also tested associations between loneliness and inflammatory stress responses using change scores (mean changes between baseline and post‐task values: 45 min post‐task minus baseline, 75 min post‐task minus baseline). Cortisol was analyzed using individual values and AUC to investigate total cortisol output across the session. Age, sex, education, marital status, smoking status, and BMI were included as covariates in all regression models. These covariates were selected as previous research suggests that these factors might influence physiological function (Jones et al., 2012; Kudielka, Buske‐Kirschbaum, Hellhammer, & Kirschbaum, 2004; Roy, Steptoe, & Kirschbaum, 1994; Steptoe et al., 2002). The analyses looking at associations of loneliness and changes in biological measures included the baseline level of the biological factor as an additional covariate.

We conducted a number of preliminary analyses to check that the associations between loneliness and biological responses did not vary as a function of participant characteristics. We previously detected a sex difference in the relationship between loneliness and stress responses in healthy individuals (Hackett et al., 2012). Loneliness was not correlated with sex in the current study. We also checked whether including sex as an interaction term would alter our results in this sample of participants with diabetes. This interaction term was not significant and did not change the pattern of results, so it was not included in our final models. Some of our participants were taking medication at the time of testing. Loneliness was not correlated with medication. We also assessed whether antidiabetic medication, β‐blockers, cholesterol medication, antihypertensive medication, or HbA1c interacted with loneliness. No significant interaction between loneliness and any medication or HbA1c was detected, so we did not include these variables in our final models. As 20% of our sample was nonwhite, we checked for an interaction with ethnicity. We detected no significant Loneliness × Ethnicity interaction, and loneliness was not significantly correlated with ethnicity, so ethnicity is not included in the models presented in Results.

Results are presented as unstandardized regression coefficients (*B*) with 95% confidence intervals (CI). Significant effects from the regression analyses are illustrated by comparing high and low loneliness groups defined by a median split using ANOVA. Raw values are presented in tables and figures for ease of interpretation. All analyses were conducted using SPSS version 24 (SPSS, Chicago, IL).

### 2.8 Sensitivity analyses

Depressed mood was measured as part of the larger study (Steptoe et al., 2014). Preliminary analyses indicated that loneliness and depressive symptoms were significantly correlated in our sample (*r = *0.454, *p* < 0.001) Therefore, in supplementary analyses, we added depression as an additional covariate to our main statistical models. Time of testing in the laboratory may be an important issue for investigations of cortisol due to its distinctive diurnal patterning (Adam et al., 2006). Therefore, we checked whether associations between cortisol and loneliness varied depending on the time of testing (am/pm).

## 3 RESULTS

### 3.1 Participant characteristics

A total of 135 people took part in the study. Of these, 101 participants had complete information for all IL‐6 and IL‐1RA measurements, and 102 individuals had full MCP‐1 data. The missing data were due to issues in blood sampling as previously reported (Panagi, Poole, Hackett, & Steptoe, 2018). Issues included difficulties maintaining a functioning cannula in obese participants and cannula failure part way through the session. For cortisol, 122 participants had complete information, in this case missing data were due to assaying issues when the samples were sent for processing. Specific *N*s are reported for models relating to these dependent variables. The participant characteristics are detailed in Table 1. Participants were aged 63.81 ± 6.93 years on average, with a range of 50–75 years. The majority of the participants were male (61.5%), married (50.4%), and of white ethnicity (80%). Most of the sample was educated to university degree level (63%) and nonsmoking (85.2%). BMI ranged from 19.20–47.80 kg/m^2^, and the average BMI was in the obese range (BMI > 30). The majority of participants were taking medication for their condition (80.3%). Levels of HbA1c were less than 6.5% in 28.7% of the sample, between 6.5%–7.5% in 38.8%, and over 7.5% in 32.6% of participants. The average loneliness score was 36.89 ± 11.83 with a range of 20–68; the 75th percentile for loneliness was a score of 45. Loneliness scores were not significantly related to age, sex, ethnicity, education, smoking, diabetic medication use, BMI, or HbA1c (*p*s > 0.087). Loneliness was significantly related to marital status (*p < *0.001), with married individuals reporting lower levels of loneliness on average (32.72 ± 8.72) than single (44.92 ± 13.05), or those divorced or separated or widowed participants (38.89 ± 12.47).

**Table 1:** Participant characteristics

Variable	Mean (*SD*) or *n* (%)	Range
Age (years)	63.81 (6.93)	50–75
Sex (% men)	83 (61.5%)	
Ethnicity (% white)	108 (80%)	
Marital status (% yes)		
Single	29 (21.5%)	
Married	68 (50.4%)	
Divorced or widowed	38 (28.1%)	
Education (%)		
No formal education	12 (8.9%)	
O‐level (Junior high)	25 (18.5%)	
A‐level (High school)	13 (9.6%)	
University degree	85 (63%)	
Smoking (% yes)	20 (14.8%)	
BMI (m^2^/kg)	30.67 (5.75)	19.20–47.80
HbA1c (%)*	7.28 (1.44)	5.40–13.10
Diabetic medication (% yes)	106 (80.3%)	
Loneliness score	36.89 (11.83)	20–68
CES‐D score^†^	11.69 (8.87)	0–43

Note

BMI = body mass index; CES‐D = Center for Epidemiologic Studies Depression scale; HbA1c = glycated hemoglobin; *SD* = standard deviation.

*N* = 135.

* *n = *129.

^†^ *n* = 132.

### 3.2 Responses to stress

Details of participants’ subjective and biological responses to stress are presented in Table 2. We found significant main effects of trial for IL‐6, MCP‐1, and cortisol and subjective stress levels (*p*s *< *0.001). There was no significant main effect of trial for IL‐1RA, *F*(2.70, 270.17) *= *0.33*, p = *0.926. IL‐6 was observed to increase following the tasks with a significant increase from baseline detected at 75 min post‐task, in keeping with previous laboratory studies (Steptoe, Hamer, & Chida, 2007). The mean increase in IL‐6 concentrations from baseline to 75 min post‐task was 0.24 ± 0.81 pg/ml. The pattern of response was different for MCP‐1, with concentrations observed to decline significantly over the session with an average decrease of 4.20 ± 19.16 pg/ml and 7.47 ± 16.54 pg/ml between baseline and 45 and 75 min post‐task, respectively. Cortisol concentrations also fell significantly immediately following the stress tasks, with an average decrease of 1.26 ± 2.65 nmol/l immediately after the tasks and 2.23 ± 3.76 nmol/l decrease 20 min later. Participant’s subjective stress levels significantly increased in response to the tasks and were observed to decrease again to baseline values during the recovery period. No significant interaction between loneliness and stress ratings over the laboratory session was detected (*p* = 0.793).

**Table 2:** Subjective, inflammatory, and neuroendocrine responses to stress

	*N*	Baseline	Immediately post‐task	20 min post‐task	45 min post‐task	75 min post‐task
IL‐6 (pg/ml)	101	2.03^a^ (1.13)	2.04^a^ (1.09)		2.14 (1.20)	2.27^b^ (1.21)
IL‐1RA (pg/ml)	101	816.96 (424.18)	817.90 (410.42)		823.78 (422.93)	820.74 (427.53)
MCP‐1 (pg/ml)	102	116.06^a ^(33.94)	114.85^a^ (38.31)		111.86 (36.17)	108.59^b^ (32.53)
Cortisol (nmol/l)	122	9.95^a^ (5.38)	8.69^b^ (4.38)	7.72 (3.96)	6.93 (4.06)	7.19 (5.56)
Subjective stress (0–)	129	1.50^a ^(0.90)	4.50^b ^(1.52)	1.57^a ^(1.06)	1.52^a ^(0.96)	1.44^a ^(0.95)

Note

Values in rows with different superscripts (^a, b^) are significantly different from one another (*p < *0.05). Values are presented as means (standard deviations). IL‐1RA = interleukin‐1 receptor antagonist; IL‐6 = interleukin‐6; MCP‐1 = monocyte chemoattractant protein‐1.

### 3.3 Loneliness and biological responses to stress

There was no association between loneliness and baseline plasma IL‐6 concentrations (*B* = 0.003, CI = −0.001 to 0.006, *p* = 0.174). Regressions on the change in IL‐6 between baseline and 45 min post‐task (*B* = 0.000, CI = −0.002 to 0.002, *p* = 0.900) and 75 min post‐task (*B* = −0.002, CI = −0.005 to 0.000, *p* = 0.055) were also nonsignificant. There were no significant associations between loneliness and raw IL‐6 values over the laboratory session (*p*s > 0.125).

Similarly, for IL‐1RA, we failed to detect an association between loneliness and baseline IL‐1RA values (*B* = 0.000, CI = −0.002 to 0.003, *p* = 0.764). Regressions on change in IL‐1RA between baseline and 45 min post‐task (*B* = 0.000, CI = −0.001 to 0.001, *p* = 0.451) and 75 min post‐task (*B* = 0.000, CI = −0.001 to 0.002, *p* = 0.392) were also nonsignificant. The analyses investigating the link between raw IL‐1RA values and loneliness were also nonsignificant (*p*s > 0.405).

**Figure 1:**
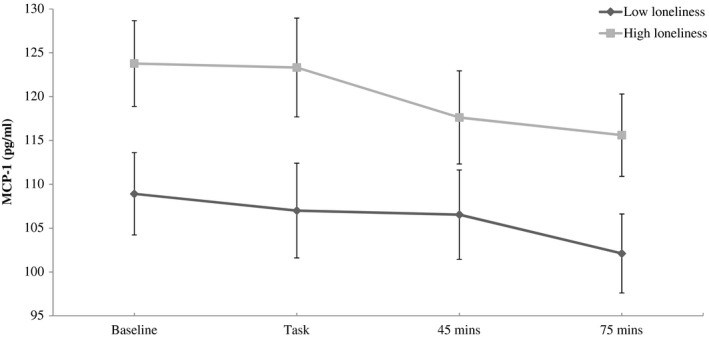
MCP‐1 responses for high and low loneliness groups over the laboratory session. Values are adjusted for age, sex, smoking, BMI, education, and marital status. Error bars are standard error of mean

For MCP‐1, there was no significant association between loneliness and baseline concentrations in the laboratory (*B* = 0.304, CI = −0.239 to 0.846, *p* = 0.270). However, there was a significant association between loneliness and raw MCP‐1 concentrations at 75 min post‐task (*B* = 0.713, CI = 0.106 to 1.321, *p* = 0.022), with higher values observed in lonelier participants. This effect was independent of baseline age, sex, education, marital status, BMI, and smoking. In unadjusted analysis, there was a significant association between loneliness and raw MCP‐1 immediately post‐task (*B* = 0.724, CI = 0.142 to 1.306, *p = *0.015) and a weaker association at 45 min post‐task (*B* = 0.551, CI = −0.012 to 1.114, *p = *0.055). However, these raw values immediately post‐task (*B* = 0.607, CI = −0.013 to 1.228, *p* = 0.055) and 45 min post‐task did not reach statistical significance (*B* = 0.525, CI = −0.079 to 1.128, *p* = 0.088) in fully adjusted models. The pattern of MCP‐1 concentrations across the laboratory session in high and low loneliness groups can be found in Figure 1. Values of MCP‐1 remained greater in the high loneliness group throughout the session. We did not detect an association between loneliness and change in MCP‐1 between baseline and 45 min and 75 min post‐task (*p*s > 0.431).

**Figure 2:**
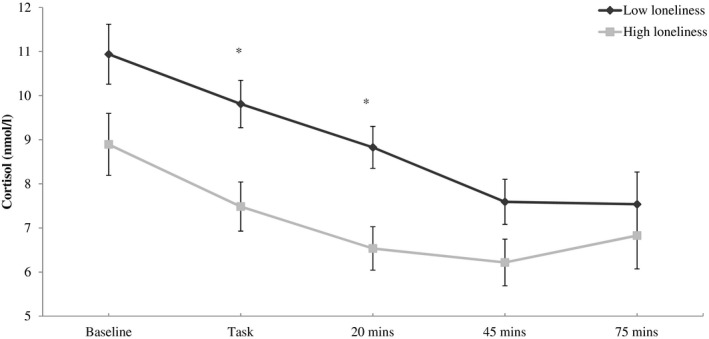
Cortisol responses for high and low loneliness groups over the laboratory session. Values are adjusted for age, sex, smoking, BMI, education, and marital status. Error bars are standard error of mean

In the analyses of cortisol, there was again no association with loneliness at baseline (*B* = −0.001, CI = −0.004 to 0.002, *p* = 0.546). However, cortisol concentrations immediately post‐task (*B* = −0.003, CI = −0.005 to −0.002, *p < *0.001), 20 min post‐task (*B* = −0.017, CI = −0.006 to −0.002, *p < *0.001) and 45 min post‐task (*B* = −0.004, CI = −0.007 to −0.001, *p* = 0.016) were lower in more lonely individuals after adjustment for covariates. The association between loneliness and cortisol was further examined using the cortisol AUC measure. There was an inverse association between loneliness and cortisol AUC (*B* = −4.429, CI = −8.109 to −0.750, *p* = 0.019). The difference in cortisol levels between participants with high and low loneliness scores is illustrated in Figure 2. Cortisol levels declined across the laboratory session in both groups. However, higher loneliness was associated with a significantly greater decrease in cortisol output over the testing period as indexed by AUC.

### 3.4 Sensitivity analyses

To explore whether the significant correlation between depression and loneliness influenced our results, we conducted some sensitivity analyses. There was no significant interaction between loneliness and depressive symptoms in the current sample. Depressive symptoms were not a significant predictor of MCP‐1 at 75 min or any other MCP‐1 measure during the laboratory session (*p*s > 0.380). When adding depressive symptoms as an additional covariate to our models, the association between loneliness and MCP‐1 at 75 min remained significant (*B* = 0.966, CI = 0.124 to 1.492, *p* = 0.021). The significant association between loneliness and cortisol immediately post‐task was also maintained (*B* = −0.002, CI = −0.004 to 0.000, *p* = 0.026). However, the associations between loneliness and cortisol values 20 min post‐task, 45 min post‐task, and cortisol AUC were attenuated (*p*s > 0.128).

We checked whether associations between cortisol and loneliness varied depending on the time of testing (am/pm). Time of testing did not influence the association between loneliness and cortisol immediately post‐task or 20 min later. However, for cortisol AUC and cortisol 45 min post‐task, differences were detected. For those tested in the afternoon, the association between loneliness and cortisol AUC and cortisol 45 min post‐task remained (*p*s *< *0.016). However, there were no significant associations for those tested in the morning (*p*s > 0.390).

## 4 DISCUSSION

This study examined the association between loneliness and inflammatory and neuroendocrine responses to an acute stress paradigm in a group of older adults with T2D. Our findings show that loneliness was not associated with IL‐6 responses to stress or IL‐1RA responses. However, we did find associations for MCP‐1 and for cortisol. Specifically, we showed that higher values of MCP‐1 were observed in lonelier participants at the 75 min post‐task recovery point. There was also a trend toward greater MCP‐1 values immediately post‐task and 45 min later for lonelier individuals. Moreover, lonelier participants also showed significantly lower cortisol concentrations immediately post‐task, and at the 20 min and 45 min post‐task recovery points. This latter finding was confirmed in analyses using the cortisol AUC measure as a measure of total cortisol output across the session, with results showing loneliness was inversely associated with cortisol AUC. Our models took into account a range of demographic, behavioral, and biological covariates, and results were upheld.

Previous work has shown loneliness to be commonly experienced in the UK (Victor & Yang, 2012), and this is supported by our results showing that 25% of our sample had a score of 45 or above out of a possible 80 on the Revised UCLA Loneliness Scale; this represents a higher score in comparison to the normative data for the scale (37.06 for male and 36.06 female individuals; Russell et al., 1980). The mean loneliness in our sample was in line with these normative values at 36.89 ± 11.83 and is consistent with previous work in the behavioral medicine field. For example, Steptoe et al., 2004, examined loneliness in a healthy subsample of the Whitehall II cohort of civil servants, finding a mean of 36.3 ± 9.5 on this scale.

Our laboratory findings can be interpreted in light of earlier research in this field. As previously mentioned, three studies have investigated the effects of loneliness on stress responses in a laboratory setting among healthy adults (Hackett et al., 2012; Jaremka et al., 2013; Steptoe et al., 2004). These studies showed loneliness to be associated with increases in fibrinogen in men and women (Steptoe et al., 2004), raised IL‐6, IL‐1RA, and MCP‐1 in women only (Hackett et al., 2012), and greater LPS stimulated IL‐6 and TNF‐α (Jaremka et al., 2013). Collectively these findings therefore suggest a heightened inflammatory response to stress among lonely adults. Our findings have added to this literature by examining these effects among those with T2D, for whom a heightened inflammatory profile has previously been observed (Pickup, 2004; Steptoe et al., 2014). We found that, among those with T2D, loneliness was associated with higher raw values in MCP‐1 during the recovery period. The association between loneliness and MCP‐1 at 75 min was robust to adjustment for covariates. There were weaker associations between loneliness and MCP‐1 immediately post‐task (*p = *0.015) and 45 min post‐task (*p = *0.055) in unadjusted analyses. No effects were found for IL‐6 or IL‐1RA post‐task scores; no effects were found for inflammatory responses to stress (i.e., the change in inflammation from baseline to recovery). Our findings do, however, lend support for the hypothesis that MCP‐1 may be a particularly important marker of distress, even among people with diabetes.

MCP‐1 is a member of the chemoattractant cytokine family and is produced after stimulation by other cytokines and is involved in the regulation of migration and infiltration of macrophages (Deshmane, Kremlev, Amini, & Sawaya, 2009). Elevated MCP‐1 has been linked with depressive symptoms (Suarez, Krishnan, & Lewis, 2003) and chronic psychosocial stress (Åsberg et al., 2009), as well as the aforementioned loneliness in women (Hackett et al., 2012). Our study suggests that it is also associated with loneliness among T2D populations following laboratory stress. More work is needed to corroborate the small effect we observed and to denote its clinical significance.

Some evidence suggests that MCP‐1 is involved in the development and progression of CVD (Damås et al., 2000; Younce & Kolattukudy, 2010), which adds to the importance of our finding since diabetes and CVD are closely related comorbid conditions (Emerging Risk Factors Collaboration et al., 2010). Loneliness has also been linked with the onset of CHD. A 2016 meta‐analysis looking at the combined effect of loneliness and social isolation found that those with poorer social relationships had a 29% increased risk of incident CHD (Valtorta et al., 2016). Therefore, our findings contribute to this growing literature suggesting MCP‐1 may represent a common biological mechanism through which loneliness in populations with diabetes could lead to an increased risk of future CVD; this hypothesis requires testing in future studies.

Despite the literature linking neuroendocrine dysfunction with T2D (Hackett et al., 2014), there has been little work investigating dynamic cortisol responses to stress in this population. Our participants were part of a larger study comparing physiological stress responses in people with T2D and healthy controls (Steptoe et al., 2014). In comparison to healthy individuals, the participants with diabetes were found to have blunted cortisol responses to stress. Previous laboratory investigations in healthy individuals have supported an association between loneliness and cortisol responses to acute stress, with Hackett et al. (2012) revealing an association between higher loneliness and reduced cortisol responsivity. Our study is the first, to our knowledge, to test these effects in a population of individuals with T2D finding congruent results such that loneliness was associated with lower cortisol concentrations immediately post‐task, and at the 20 min and 45 min post‐task recovery points, and with lower total cortisol output across the session. In the context of our earlier work demonstrating that participants with T2D have blunted cortisol responses to stress, it is possible that loneliness acts to compound these blunted stress responses. Lower responses to cortisol may reflect the burnout component of allostatic load theory (McEwen, 1998), with participants unable to mount an appropriate response to challenge. Taken together with the MCP‐1 results, our findings suggest lonelier individuals may have insufficient GR signaling to inhibit inflammation following stress; mechanistic work of this type would help tease out these biological pathways in greater detail.

Other mechanistic pathways linking loneliness with poor health outcomes must also be acknowledged. Negative health behaviors are well‐established risk factors for poor health outcomes and are also known to be associated with loneliness. For example, in a study of over 8,000 adults from the English Longitudinal Study of Ageing (ELSA) cohort, loneliness was associated with an increased likelihood of smoking and being inactive (Shankar et al., 2011). Another analysis of the ELSA data found that lonelier individuals were more likely to be obese than their less lonely counterparts (Whisman, 2010). However, most studies investigating the link between loneliness and future ill‐health have controlled for health behaviors (Holt‐Lunstad et al., 2015; Thurston & Kubzansky, 2009), strengthening our position that direct biological pathways are also involved. Indeed, we controlled for smoking and BMI in our models, and our findings were upheld.

Strengths of this study include the recruitment of a population sample of men and women with well‐documented T2D who were free of cardiovascular complications. We examined three inflammatory markers and cortisol, and their patterns of stress responsivity using a standard stress protocol. We included a long post‐task blood sampling period of 75 min to ensure that we captured delayed inflammatory responses. We obtained detailed medication data and took this into account in the analyses. Limitations also need to be taken into account. We excluded those with current clinical mood disorders from the study. As depression is common in people with diabetes (Anderson, Freedland, Clouse, & Lustman, 2001) and associated with loneliness (Weeks, Michela, Peplau, & Bragg, 1980), this reduces the generalizability of our findings. This exclusion may have led us to underestimate levels of loneliness in those with T2D. Loneliness is associated with negative health behaviors (Shankar et al., 2011) including sleep and exercise. These behaviors were not taken into consideration in the current study. We were able to collect full biological data on only about three quarters of participants due to difficulties in blood sampling. Our stress tasks provoked robust cardiovascular responses (data not shown; see Steptoe et al., 2014) and significant increases in subjective stress. However, social evaluative tasks are suggested to elicit greater cortisol responses than the tasks used in the current study (Dickerson & Kemeny, 2004). It is plausible that different effects may have been observed using more distressing tasks. Laboratory responses were tested on only one occasion. Causal relationships between greater loneliness and biological stress responses cannot therefore be drawn. Longitudinal research would also help elucidate the extent to which chronic loneliness and changes in loneliness over time are associated with inflammatory and neuroendocrine biomarkers. Participants of this study were middle‐aged men and women with T2D and without a history of CHD. They were recruited from the London area and most of them were of white European ethnicity, thus we do not know how far the results generalize to other cohorts.

In conclusion, we report findings from a laboratory stress study of older T2D participants revealing lonelier individuals had higher 75‐min post‐stress MCP‐1 values and reduced cortisol output at immediate, 20 min, and 45 min post‐stress recovery. There was a trend toward lower MCP‐1 values immediately post‐task and 45 min later in lonelier individuals with T2D. Our findings point to important biological mechanisms linking loneliness with adverse health outcomes, though further work is needed to tease apart the exact biological mechanisms of this effect.
